# Single-crystal X-ray diffraction study of a host–guest system comprising monofunctionalized-hydroxy pillar[5]arene and 1-octa­namine

**DOI:** 10.1107/S2056989018010034

**Published:** 2018-07-20

**Authors:** Talal F. Al-Azemi, Mickey Vinodh, Abdirahman A. Mohamod, Fatemeh H. Alipour

**Affiliations:** a Department of Chemistry, Kuwait University, PO Box 5969, Safat 13060, Kuwait

**Keywords:** Functionalized pillararene, host–guest inter­action, encapsulation, crystal structure

## Abstract

Co-crystallization of a monofunctionalized hy­droxy pillar[5]arene with 1-octa­namine resulted in the formation of an inclusion complex where the alkyl chain is threaded in the macrocycle cavity, namely 1,2,3,4-(1,4-dimeth­oxy)-5-(1-hy­droxy-4-meth­oxy)-pillar[5]arene–1-octa­namine–water (1/1/1), C_44_H_48_O_10_·C_8_H_19_N·H_2_O. The guest compound is stabilized inside the cavity by hydrogen-bonding and C—H⋯π inter­actions. The water mol­ecule in the asymmetric unit mediates the formation of a supra­molecular dimer by hydrogen-bonding inter­actions. These functionalized-pillararene hosts expand the possibility of exploring more supra­molecular inter­actions with various guest species.

## Abstract 2

The crystal structure and supra­molecular inter­actions between a hy­droxy-functionalized pillarene and 1-octa­namine guest mol­ecule, which forms an inter­esting host–guest system, are reported.

## Chemical context   

Pillar[5]arenes are a relatively new class of three-dimensional macrocyclic compounds having a well-defined inner cavity for guest encapsulation. Unlike cone-shaped calixarene or resorcinarene-type structures, the pillararenes have a tabular cavity, which makes them inter­esting mol­ecular hosts. It is well known that pillar[5]arenes exhibit an outstanding ability to selectively bind different kinds of guest mol­ecules and thus are valuable chemical entities in the areas of host–guest systems and mol­ecular recognition (Ogoshi *et al.*, 2008 ▸). The guest moieties that could be encapsulated by pillararenes include both neutral and charged guest species and the preference will be for those having long alkyl chains. Appropriate function­alization of the pillararene framework could enable efficient control over the binding properties of these macrocycles with a variety of guest species (Han *et al.*, 2010 ▸, 2015 ▸; Pan & Xue, 2013 ▸; Hu *et al.*, 2016 ▸).

Chemical modification of the pillararene system could be achieved in two ways, namely cyclization of appropriately functionalized monomers or functionalization of preformed pillararenes (Al-Azemi *et al.*, 2017 ▸). In the former, co-cyclization of pre-functionalized monomers in an appropriate feed ratio could be employed to generate pillararenes having the desired functionalities in terms of numbers and positions.

The pillar[5]rene system having one hy­droxy group is inter­esting because this OH– function is susceptible for further chemical transformation (Al-Azemi et *al.*, 2018). Furthermore, the OH– group in pillararenes could involve hydrogen bonding with guest mol­ecules and/or with neighboring pillararenes, which makes them valuable compounds in mol­ecular recognition and supra­molecular chemistry. We have recently reported details of the host–guest complexation between mono-hy­droxy-pillar[5]arenes with long-chain alkyl alcohol guests (Al-Azemi et *al.*, 2018). It was observed that the encapsulation characteristics of the pillar[5]arene was affected by the presence of the hy­droxy group, resulting in the formation of a 1:2 complex with long-chain alkyl alcohols.
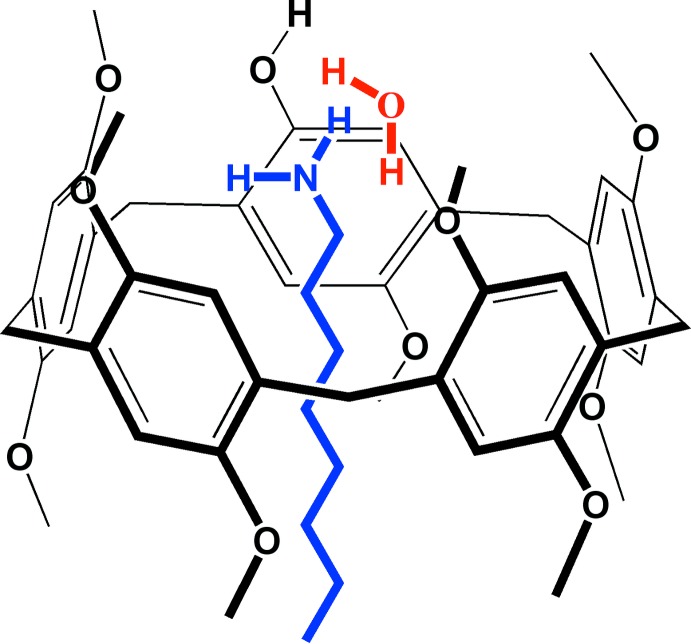


In this work we report the crystal structure of the inclusion complex consisting of 1-(1-hy­droxy-4-meth­oxy)-2,3,4,5-(1,4-dimeth­oxy)-pillar[5]arene (**Pil-OH)** and 1-octa­namine (**OctNH_2_**). The structural features and supra­molecular host–guest inter­actions of this co-crystalline system (**Pil-OH·OctNH_2_**) has been addressed and discussed.

## Structural commentary   

**Figure 1:**
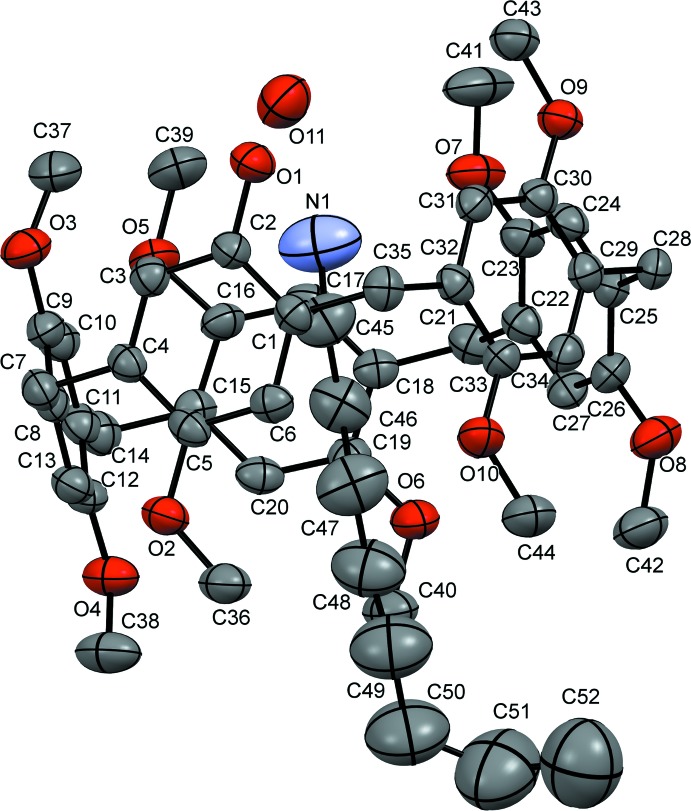
Displacement ellipsoid representation (30% probability) of **Pil-OH·OctNH_2_**. Hydrogen atoms are omitted for clarity.

The crystal structure of the inclusion complex **Pil-OH·OctNH_2_** is given in Fig. 1 ▸. The mono-hy­droxy-pillar[5]arene (**Pil-OH)** has a rigid three-dimensional macrocyclic architecture with a wide cavity having a penta­gonal shape. The 1-octa­namine mol­ecule is threaded inside the pillararene cavity and one water is included in asymmetric unit, displaying strong hydrogen-bonding inter­actions with the amino group of the guest mol­ecule inside the cavity and the hy­droxy group on the pillararene system *via* O11—H11*A*⋯N1 and O11—H11*B*⋯O1 bonds respectively (Table 1 ▸).

## Supra­molecular features   

**Figure 2:**
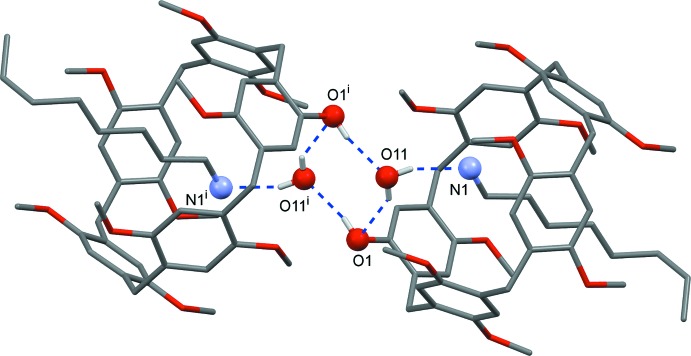
Hydrogen-bonding inter­actions between **Pil-OH·OctNH_2_** systems showing the formation of a water-mol­ecule-mediated supra­molecular dimer. [Symmetry code: (i) −*x* + 2, −*y* + 1, −*z* + 1.]

**Figure 3:**
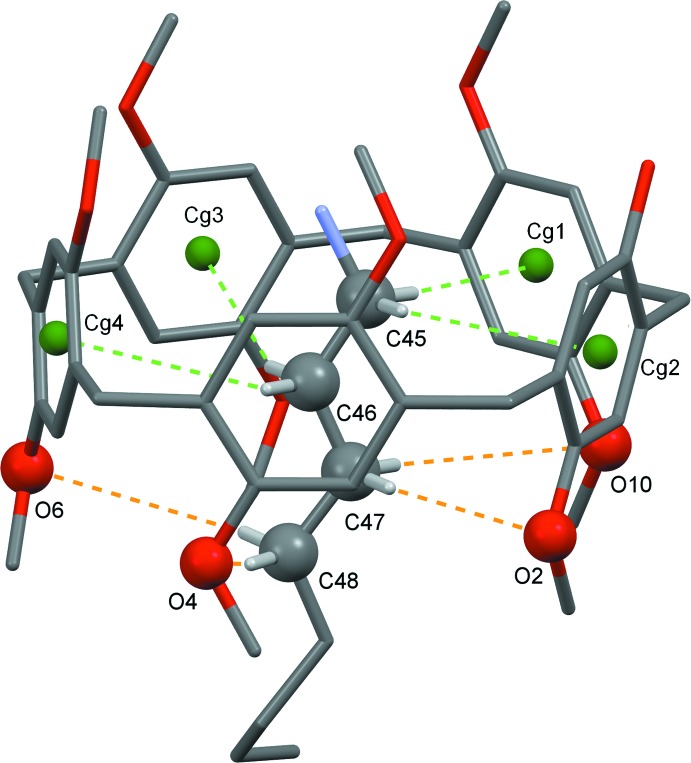
Crystal structure of the inclusion complex **Pil-OH·OctNH_2_** showing weak C—H⋯O and C—H⋯ π inter­actions where *Cg*1–4 are the centroids of the aromatic rings in the pillararene system. C—H⋯O inter­actions are represented as brown and C—H⋯ π as green dashed lines.

In the title inclusion complex, the water mol­ecule mediates the formation of supra­molecular dimers through O1^i^—H1^i^⋯O11[symmetry code: (i) −*x* + 2, −*y* + 1, −*z* + 1] and O11—H11⋯O1 hydrogen-bonding inter­actions (Table 1 ▸), as illustrated in Fig. 2 ▸. In addition, the encapsulated 1-octa­namine is stabilized inside the cavity by C—H⋯π inter­actions with the pillararene aromatic ring and C—H⋯O inter­actions at the meth­oxy groups on the rim of the macrocycle, which act as hydrogen-bond acceptors. These weak inter­actions are shown in Fig. 3 ▸ and the corresponding inter­action distances are given in Table 2 ▸. The threaded terminal methyl group of the alkyl chain of the 1-octa­namine guest is positioned outside the pillararene moiety where it engages in a weak inter­molecular C—H⋯O inter­action with the meth­oxy group of another pillararene mol­ecule [C52—H52*C*⋯O7^ii^; symmetry code: (ii) *x* − 1, *y*, *z*]. A weak C—H⋯O type pillararene–pillararene inter­action is also observed [C44—H44*B*⋯O3^iii^; symmetry code: (iii) *x* − 1, *y* − 1, *z*].

## Synthesis and crystallization   

The synthesis of 1-(1-hy­droxy-4-meth­oxy)-2,3,4,5-(1,4-di­meth­oxy)pillar[5]arene has been reported previously (Al-Azemi *et al.*, 2018 ▸). The co-crystallization of pillararene with 1-octa­namine was undertaken by adding pillararene (20 mg) and 1-octa­namine (50 µL) to chloro­form (0.5 mL) in a small vial, followed by a very slow solvent evaporation. Within six days, crystals of a suitable size for diffraction analysis had formed.

## Refinement   

Crystal data, data collection and structure refinement details are summarized in Table 3 ▸. The hydrogen atoms belonging to water, the OH fraction of the pillarene apex and the NH_2_ group of 1-octa­namine were found in the electron density map and freely refined. All other hydrogen atoms are placed at calculated positions and refined using a riding model: C—H = 0.95–0.99 Å with *U*_iso_(H) = 1.2*U*_eq_(C).

## Supplementary Material

Crystal structure: contains datablock(s) I. DOI: 10.1107/S2056989018010034/dx2005sup1.cif

Structure factors: contains datablock(s) I. DOI: 10.1107/S2056989018010034/dx2005Isup4.hkl

CCDC reference: 1855261

Additional supporting information:  crystallographic information; 3D view; checkCIF report

## Figures and Tables

**Table 1:** Hydrogen-bond geometry (Å, °)

*D*—H⋯*A*	*D*—H	H⋯*A*	*D*⋯*A*	*D*—H⋯*A*
O11—H11*A*⋯N1	0.83 (2)	1.98 (5)	2.770 (10)	159 (7)
O11—H11*B*⋯O1	0.80 (10)	2.40 (10)	3.060 (10)	145 (10)
O1—H1⋯O11^i^	0.82 (5)	1.90 (5)	2.711 (7)	168 (9)

Symmetry code: (i) 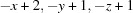.

**Table 2:** Summary of weak inter­actions (C—H⋯π and C—H⋯O; Å, °) between the pillararene and 1-octa­namine mol­ecules *Cg*1, *Cg*2, *Cg*3 and *Cg*4 are the centroids of the C1–C6, C29–C34, C15–C20 and C22–C27 rings, respectively.

*D*—H⋯*A*	H⋯*A*	*D*⋯*A*	*D*—H⋯*A*
C45—H45*A*⋯*Cg*1	3.02	3.815 (10)	139
C45—H45*B*⋯*Cg*2	2.89	3.867 (8)	175
C46—H46*A*⋯*Cg*3	3.10	3.790 (9)	128
C46—H46*B*⋯*Cg*4	3.18	4.106 (12)	157
C47—H47*A*⋯O2	3.10	4.070 (13)	166
C47—H47*B*⋯O10	3.26	4.158 (10)	151
C48—H48*A*⋯O4	3.17	4.094 (12)	156
C48—H48*B*⋯O6	3.24	3.974 (14)	132
C52—H52*C*⋯O7^ii^	2.43	3.39 (2)	168
C44—H44*B*⋯O3^ii^	2.65	3.611 (8)	167

Symmetry codes: (ii) *x* − 1, *y*, *z*; (iii) *x* − 1, *y* − 1, *z*.

**Table 3:** Experimental details

Crystal data	
Chemical formula	C_44_H_48_O_10_·C_8_H_19_N·H_2_O
*M*_r_	884.08
Crystal system, space group	Triclinic, *P* 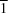
Temperature (K)	150
*a*, *b*, *c* (Å)	12.147 (12), 12.341 (12), 19.406 (19)
α, β, γ (°)	91.433 (11), 90.181 (11), 119.182 (9)
*V* (Å^3^)	2539 (4)
*Z*	2
Radiation type	Mo *K*α
μ (mm^−1^)	0.08
Crystal size (mm)	0.12 × 0.11 × 0.02

Data collection	
Diffractometer	Rigaku R-AXIS RAPID
Absorption correction	Multi-scan (*ABSCOR*; Higashi, 1995 ▸)
*T*_min_, *T*_max_	0.000, 0.998
No. of measured, independent and observed [*I* > 2σ(*I*)] reflections	19245, 8604, 3452
*R*_int_	0.078
(sin θ/λ)_max_ (Å^−1^)	0.589

Refinement	
*R*[*F* ^2^ > 2σ(*F* ^2^)], *wR*(*F* ^2^), *S*	0.094, 0.349, 0.95
No. of reflections	8604
No. of parameters	606
No. of restraints	44
H-atom treatment	H atoms treated by a mixture of independent and constrained refinement
Δρ_max_, Δρ_min_ (e Å^−3^)	0.36, −0.27

Computer programs: *CrystalClear-SM Expert* (Rigaku, 2009 ▸); *CrystalStructure* (Rigaku, 2010 ▸), *Il Milione* (Burla *et al.*, 2007 ▸), *SHELXL2017* (Sheldrick, 2015 ▸); *ShelXle* (Hübschle *et al.*, 2011 ▸) and *Mercury* (Macrae *et al.*, 2006 ▸).

## supplementary crystallographic information

### Crystal data

**Table:**

C_44_H_48_O_10_·C_8_H_19_N·H_2_O	*Z* = 2
*M**_r_* = 884.08	*F*(000) = 952
Triclinic, *P*1	*D*_x_ = 1.157 Mg m^−^^3^
*a* = 12.147 (12) Å	Mo *K*α radiation, λ = 0.71075 Å
*b* = 12.341 (12) Å	Cell parameters from 1156 reflections
*c* = 19.406 (19) Å	θ = 3.2–24.5°
α = 91.433 (11)°	µ = 0.08 mm^−^^1^
β = 90.181 (11)°	*T* = 150 K
γ = 119.182 (9)°	Platelet, colorless
*V* = 2539 (4) Å^3^	0.12 × 0.11 × 0.02 mm

### Data collection

**Table:**

Rigaku R-AXIS RAPID diffractometer	3452 reflections with *I* > 2σ(*I*)
Detector resolution: 10.000 pixels mm^-1^	*R*_int_ = 0.078
ω scans	θ_max_ = 24.8°, θ_min_ = 3.2°
Absorption correction: multi-scan (ABSCOR; Higashi, 1995)	*h* = −14→14
*T*_min_ = 0.000, *T*_max_ = 0.998	*k* = −14→14
19245 measured reflections	*l* = −22→22
8604 independent reflections	

### Refinement

**Table:**

Refinement on *F*^2^	44 restraints
Least-squares matrix: full	Hydrogen site location: mixed
*R*[*F*^2^ > 2σ(*F*^2^)] = 0.094	H atoms treated by a mixture of independent and constrained refinement
*wR*(*F*^2^) = 0.349	*w* = 1/[σ^2^(*F*_o_^2^) + (0.2*P*)^2^] where *P* = (*F*_o_^2^ + 2*F*_c_^2^)/3
*S* = 0.95	(Δ/σ)_max_ = 0.006
8604 reflections	Δρ_max_ = 0.36 e Å^−^^3^
606 parameters	Δρ_min_ = −0.27 e Å^−^^3^

### Special details

**Table:**

Geometry. All esds (except the esd in the dihedral angle between two l.s. planes) are estimated using the full covariance matrix. The cell esds are taken into account individually in the estimation of esds in distances, angles and torsion angles; correlations between esds in cell parameters are only used when they are defined by crystal symmetry. An approximate (isotropic) treatment of cell esds is used for estimating esds involving l.s. planes.

### Fractional atomic coordinates and isotropic or equivalent isotropic displacement parameters (Å^2^)

**Table:**

	*x*	*y*	*z*	*U*_iso_*/*U*_eq_	
O1	0.7998 (4)	0.4416 (4)	0.5308 (2)	0.0863 (12)	
H1	0.873 (4)	0.497 (8)	0.537 (6)	0.26 (6)*	
H1B	0.8643 (19)	0.2821 (17)	0.283 (3)	0.9 (3)*	
H1A	0.926 (2)	0.402 (4)	0.266 (2)	1.075*	
O2	0.5570 (4)	0.6085 (3)	0.35282 (19)	0.0828 (11)	
O3	1.0480 (4)	0.7681 (4)	0.3574 (2)	0.0900 (12)	
O4	0.7543 (4)	0.6894 (4)	0.1205 (2)	0.0960 (12)	
O5	1.1318 (4)	0.5864 (4)	0.1524 (2)	0.1013 (13)	
O6	0.6840 (4)	0.2542 (4)	0.0134 (2)	0.0930 (12)	
O7	0.9401 (4)	0.1253 (4)	0.1904 (2)	0.1032 (14)	
O8	0.4150 (4)	−0.0913 (4)	0.1898 (2)	0.0999 (13)	
O9	0.7136 (3)	0.0036 (3)	0.41713 (19)	0.0793 (10)	
O10	0.3518 (4)	0.1570 (3)	0.3987 (2)	0.0856 (11)	
O11	0.9620 (6)	0.3882 (5)	0.4297 (4)	0.1282 (17)	
H11A	0.952 (8)	0.382 (8)	0.3873 (8)	0.14 (4)*	
H11B	0.895 (6)	0.381 (14)	0.444 (4)	0.36 (10)*	
N1	0.8665 (8)	0.3545 (9)	0.2964 (4)	0.187 (3)	
C1	0.6093 (5)	0.4038 (4)	0.4726 (2)	0.0616 (13)	
C2	0.7389 (5)	0.4832 (4)	0.4870 (3)	0.0646 (13)	
C3	0.8014 (5)	0.5997 (4)	0.4566 (2)	0.0657 (13)	
H3	0.888107	0.652169	0.467812	0.079*	
C4	0.7446 (5)	0.6424 (4)	0.4117 (2)	0.0619 (13)	
C5	0.6149 (5)	0.5637 (4)	0.3974 (2)	0.0648 (13)	
C6	0.5476 (5)	0.4457 (5)	0.4282 (3)	0.0663 (13)	
H6	0.460041	0.394896	0.418505	0.080*	
C7	0.8171 (5)	0.7667 (4)	0.3762 (3)	0.0717 (14)	
H7A	0.761602	0.803975	0.371036	0.086*	
H7B	0.890363	0.824020	0.405532	0.086*	
C8	0.8632 (5)	0.7517 (4)	0.3053 (3)	0.0653 (13)	
C9	0.9770 (5)	0.7500 (4)	0.2978 (3)	0.0703 (14)	
C10	1.0164 (5)	0.7319 (4)	0.2321 (3)	0.0711 (14)	
H10	1.095015	0.733265	0.227972	0.085*	
C11	0.9414 (5)	0.7118 (4)	0.1728 (3)	0.0671 (14)	
C12	0.8273 (5)	0.7131 (4)	0.1804 (3)	0.0651 (13)	
C13	0.7897 (5)	0.7325 (4)	0.2446 (3)	0.0723 (14)	
H13	0.711909	0.732931	0.248182	0.087*	
C14	0.9834 (5)	0.6858 (5)	0.1028 (3)	0.0783 (16)	
H14A	0.944839	0.710599	0.065626	0.094*	
H14B	1.076111	0.737002	0.099692	0.094*	
C15	0.9459 (5)	0.5487 (5)	0.0917 (3)	0.0677 (13)	
C16	1.0202 (5)	0.4986 (5)	0.1180 (3)	0.0708 (14)	
C17	0.9803 (5)	0.3735 (5)	0.1099 (3)	0.0775 (16)	
H17	1.030774	0.342342	0.129389	0.093*	
C18	0.8681 (5)	0.2895 (5)	0.0741 (2)	0.0645 (13)	
C19	0.7951 (5)	0.3381 (5)	0.0473 (3)	0.0673 (13)	
C20	0.8359 (5)	0.4660 (5)	0.0550 (2)	0.0698 (14)	
H20	0.786551	0.497525	0.034485	0.084*	
C21	0.8264 (5)	0.1518 (5)	0.0693 (3)	0.0744 (15)	
H21A	0.901999	0.141389	0.065517	0.089*	
H21B	0.774835	0.115675	0.026728	0.089*	
C22	0.7501 (5)	0.0799 (4)	0.1308 (3)	0.0659 (13)	
C23	0.8092 (5)	0.0688 (5)	0.1907 (3)	0.0726 (14)	
C24	0.7356 (5)	0.0045 (5)	0.2471 (3)	0.0703 (14)	
H24	0.777006	−0.003112	0.286797	0.084*	
C25	0.6053 (5)	−0.0481 (4)	0.2471 (3)	0.0613 (12)	
C26	0.5478 (5)	−0.0366 (4)	0.1869 (3)	0.0703 (14)	
C27	0.6189 (5)	0.0249 (4)	0.1303 (3)	0.0690 (14)	
H27	0.576768	0.029565	0.090089	0.083*	
C28	0.5313 (5)	−0.1097 (4)	0.3107 (3)	0.0677 (14)	
H28A	0.568733	−0.155795	0.332925	0.081*	
H28B	0.443199	−0.169888	0.297094	0.081*	
C29	0.5322 (5)	−0.0127 (4)	0.3623 (3)	0.0620 (13)	
C30	0.6251 (5)	0.0447 (4)	0.4141 (3)	0.0644 (13)	
C31	0.6250 (5)	0.1358 (4)	0.4595 (3)	0.0625 (13)	
H31	0.688553	0.172627	0.494462	0.075*	
C32	0.5330 (5)	0.1733 (4)	0.4541 (3)	0.0623 (13)	
C33	0.4413 (5)	0.1172 (4)	0.4021 (3)	0.0639 (13)	
C34	0.4409 (5)	0.0264 (4)	0.3580 (3)	0.0667 (14)	
H34	0.376598	−0.010753	0.323403	0.080*	
C35	0.5383 (5)	0.2750 (4)	0.5031 (3)	0.0696 (14)	
H35A	0.451230	0.256429	0.513752	0.084*	
H35B	0.580257	0.274780	0.546904	0.084*	
C36	0.4254 (6)	0.5339 (6)	0.3381 (3)	0.0957 (19)	
H36A	0.408099	0.451973	0.320346	0.115*	
H36B	0.399726	0.574028	0.303425	0.115*	
H36C	0.377979	0.524540	0.380332	0.115*	
C37	1.1307 (6)	0.7168 (6)	0.3582 (4)	0.112 (2)	
H37A	1.081350	0.626170	0.353112	0.134*	
H37B	1.178058	0.739408	0.402066	0.134*	
H37C	1.189692	0.749539	0.320081	0.134*	
C38	0.6446 (7)	0.7030 (7)	0.1244 (4)	0.123 (3)	
H38A	0.612310	0.700893	0.077804	0.148*	
H38B	0.666172	0.782534	0.147599	0.148*	
H38C	0.579948	0.635015	0.150610	0.148*	
C39	1.2011 (6)	0.5426 (7)	0.1908 (4)	0.123 (3)	
H39A	1.272954	0.612981	0.214202	0.147*	
H39B	1.231752	0.499850	0.159669	0.147*	
H39C	1.146332	0.484752	0.225219	0.147*	
C40	0.6167 (6)	0.2999 (6)	−0.0255 (4)	0.108 (2)	
H40A	0.673850	0.360864	−0.057917	0.130*	
H40B	0.582687	0.339461	0.005726	0.130*	
H40C	0.547291	0.230861	−0.051165	0.130*	
C41	1.0011 (6)	0.1111 (8)	0.2487 (4)	0.148 (4)	
H41A	1.092620	0.162445	0.244320	0.178*	
H41B	0.979315	0.023797	0.251913	0.178*	
H41C	0.973832	0.137279	0.290344	0.178*	
C42	0.3518 (6)	−0.0589 (6)	0.1393 (4)	0.111 (2)	
H42A	0.261287	−0.100430	0.148697	0.133*	
H42B	0.365018	−0.085150	0.093413	0.133*	
H42C	0.385380	0.031267	0.140919	0.133*	
C43	0.8110 (6)	0.0576 (6)	0.4702 (3)	0.0908 (17)	
H43A	0.866291	0.145762	0.461515	0.109*	
H43B	0.860600	0.014439	0.469794	0.109*	
H43C	0.771997	0.049224	0.515331	0.109*	
C44	0.2507 (6)	0.0996 (6)	0.3489 (4)	0.106 (2)	
H44A	0.198668	0.140194	0.351356	0.128*	
H44B	0.198738	0.011365	0.358742	0.128*	
H44C	0.285413	0.107841	0.302639	0.128*	
C45	0.7525 (8)	0.3574 (8)	0.2971 (4)	0.122 (2)	
H45A	0.769361	0.436453	0.320385	0.146*	
H45B	0.693821	0.289613	0.326564	0.146*	
C46	0.6862 (9)	0.3467 (8)	0.2329 (4)	0.127 (3)	
H46A	0.743711	0.418069	0.205191	0.152*	
H46B	0.676467	0.271179	0.208279	0.152*	
C47	0.5660 (9)	0.3407 (9)	0.2301 (5)	0.151 (3)	
H47A	0.574930	0.416845	0.253525	0.181*	
H47B	0.507509	0.269708	0.257773	0.181*	
C48	0.5038 (11)	0.3280 (11)	0.1620 (5)	0.167 (3)	
H48A	0.557335	0.404621	0.136891	0.201*	
H48B	0.505917	0.258918	0.136166	0.201*	
C49	0.3773 (12)	0.3068 (13)	0.1576 (6)	0.204 (4)	
H49A	0.374764	0.375760	0.183428	0.244*	
H49B	0.323332	0.229943	0.182388	0.244*	
C50	0.3154 (13)	0.2945 (13)	0.0869 (6)	0.231 (4)	
H50A	0.275598	0.347951	0.089220	0.278*	
H50B	0.384862	0.332588	0.053709	0.278*	
C51	0.2189 (15)	0.1723 (13)	0.0547 (8)	0.284 (6)	
H51A	0.268135	0.146093	0.024831	0.341*	
H51B	0.169054	0.193908	0.023020	0.341*	
C52	0.1227 (15)	0.0544 (14)	0.0877 (9)	0.293 (7)	
H52A	0.165112	0.028166	0.121064	0.439*	
H52B	0.080349	−0.011013	0.052049	0.439*	
H52C	0.060180	0.069646	0.111430	0.439*	

### Atomic displacement parameters (Å^2^)

**Table:**

	*U*^11^	*U*^22^	*U*^33^	*U*^12^	*U*^13^	*U*^23^
O1	0.082 (3)	0.084 (2)	0.094 (3)	0.040 (2)	−0.007 (2)	0.024 (2)
O2	0.090 (3)	0.090 (2)	0.084 (3)	0.056 (2)	−0.009 (2)	0.008 (2)
O3	0.081 (3)	0.106 (3)	0.077 (3)	0.042 (2)	−0.021 (2)	−0.002 (2)
O4	0.101 (3)	0.128 (3)	0.082 (3)	0.074 (3)	−0.016 (2)	−0.003 (2)
O5	0.076 (3)	0.101 (3)	0.130 (4)	0.045 (2)	−0.020 (3)	0.003 (3)
O6	0.099 (3)	0.097 (3)	0.090 (3)	0.053 (2)	−0.031 (2)	−0.006 (2)
O7	0.072 (3)	0.140 (3)	0.103 (3)	0.054 (3)	0.004 (2)	0.041 (3)
O8	0.074 (3)	0.113 (3)	0.093 (3)	0.031 (2)	−0.015 (2)	0.008 (2)
O9	0.084 (3)	0.083 (2)	0.083 (3)	0.051 (2)	−0.013 (2)	−0.0055 (19)
O10	0.074 (3)	0.087 (2)	0.103 (3)	0.046 (2)	−0.010 (2)	−0.005 (2)
O11	0.101 (4)	0.122 (4)	0.140 (6)	0.038 (3)	−0.012 (4)	0.000 (4)
N1	0.177 (8)	0.276 (11)	0.143 (7)	0.138 (8)	−0.019 (6)	−0.021 (7)
C1	0.071 (4)	0.062 (3)	0.060 (3)	0.038 (3)	0.005 (3)	−0.001 (2)
C2	0.070 (4)	0.060 (3)	0.064 (3)	0.032 (3)	−0.005 (3)	0.005 (2)
C3	0.068 (3)	0.064 (3)	0.063 (3)	0.031 (3)	−0.001 (3)	−0.001 (2)
C4	0.077 (4)	0.058 (3)	0.058 (3)	0.039 (3)	−0.003 (3)	−0.002 (2)
C5	0.090 (4)	0.068 (3)	0.055 (3)	0.053 (3)	−0.002 (3)	−0.002 (2)
C6	0.067 (3)	0.077 (3)	0.064 (3)	0.043 (3)	0.000 (3)	−0.006 (3)
C7	0.087 (4)	0.058 (3)	0.073 (4)	0.038 (3)	−0.005 (3)	0.002 (2)
C8	0.085 (4)	0.050 (3)	0.059 (3)	0.031 (3)	−0.001 (3)	0.007 (2)
C9	0.065 (3)	0.062 (3)	0.073 (4)	0.023 (3)	−0.011 (3)	0.008 (3)
C10	0.066 (3)	0.072 (3)	0.070 (4)	0.030 (3)	0.003 (3)	0.014 (3)
C11	0.080 (4)	0.061 (3)	0.059 (3)	0.033 (3)	0.001 (3)	0.010 (2)
C12	0.074 (4)	0.069 (3)	0.057 (3)	0.038 (3)	−0.010 (3)	0.004 (2)
C13	0.079 (4)	0.073 (3)	0.073 (4)	0.044 (3)	−0.003 (3)	0.010 (3)
C14	0.080 (4)	0.078 (3)	0.075 (4)	0.036 (3)	0.007 (3)	0.016 (3)
C15	0.061 (3)	0.077 (3)	0.061 (3)	0.030 (3)	0.010 (3)	0.014 (3)
C16	0.052 (3)	0.081 (4)	0.075 (4)	0.029 (3)	−0.003 (3)	0.008 (3)
C17	0.075 (4)	0.098 (4)	0.077 (4)	0.055 (3)	0.011 (3)	0.017 (3)
C18	0.067 (3)	0.081 (3)	0.049 (3)	0.038 (3)	0.006 (3)	0.005 (2)
C19	0.071 (4)	0.076 (3)	0.053 (3)	0.035 (3)	−0.006 (3)	0.002 (3)
C20	0.072 (4)	0.092 (4)	0.055 (3)	0.046 (3)	0.006 (3)	0.016 (3)
C21	0.086 (4)	0.086 (4)	0.067 (4)	0.054 (3)	0.006 (3)	0.003 (3)
C22	0.081 (4)	0.067 (3)	0.059 (3)	0.043 (3)	0.001 (3)	−0.001 (2)
C23	0.071 (4)	0.079 (3)	0.077 (4)	0.044 (3)	0.002 (3)	0.006 (3)
C24	0.083 (4)	0.074 (3)	0.064 (3)	0.046 (3)	−0.006 (3)	0.008 (3)
C25	0.064 (3)	0.055 (3)	0.065 (3)	0.028 (2)	−0.008 (3)	−0.006 (2)
C26	0.060 (3)	0.063 (3)	0.078 (4)	0.022 (3)	−0.015 (3)	−0.007 (3)
C27	0.084 (4)	0.070 (3)	0.051 (3)	0.037 (3)	−0.012 (3)	−0.008 (2)
C28	0.069 (3)	0.048 (2)	0.076 (4)	0.021 (2)	−0.011 (3)	−0.004 (2)
C29	0.060 (3)	0.053 (3)	0.064 (3)	0.021 (2)	0.005 (3)	0.012 (2)
C30	0.063 (3)	0.064 (3)	0.067 (3)	0.031 (3)	0.002 (3)	0.010 (3)
C31	0.061 (3)	0.058 (3)	0.058 (3)	0.022 (2)	−0.003 (2)	0.007 (2)
C32	0.064 (3)	0.057 (3)	0.065 (3)	0.028 (2)	0.011 (3)	0.014 (2)
C33	0.055 (3)	0.060 (3)	0.072 (4)	0.025 (2)	−0.002 (3)	0.004 (3)
C34	0.061 (3)	0.058 (3)	0.067 (3)	0.018 (2)	−0.006 (2)	0.007 (3)
C35	0.071 (3)	0.069 (3)	0.065 (3)	0.031 (3)	0.010 (3)	0.004 (3)
C36	0.100 (5)	0.109 (4)	0.095 (5)	0.065 (4)	−0.023 (4)	−0.005 (4)
C37	0.101 (5)	0.111 (5)	0.120 (6)	0.048 (4)	−0.038 (4)	0.008 (4)
C38	0.130 (6)	0.174 (7)	0.110 (6)	0.110 (6)	−0.029 (5)	−0.014 (5)
C39	0.086 (5)	0.139 (6)	0.143 (7)	0.055 (5)	−0.029 (5)	−0.002 (5)
C40	0.113 (5)	0.126 (5)	0.103 (5)	0.072 (4)	−0.035 (4)	−0.015 (4)
C41	0.077 (5)	0.207 (8)	0.151 (7)	0.058 (5)	−0.008 (5)	0.080 (6)
C42	0.079 (4)	0.128 (5)	0.119 (6)	0.045 (4)	−0.024 (4)	0.000 (4)
C43	0.087 (4)	0.098 (4)	0.101 (5)	0.056 (3)	−0.014 (4)	−0.006 (3)
C44	0.088 (5)	0.118 (5)	0.121 (6)	0.056 (4)	−0.023 (4)	−0.002 (4)
C45	0.114 (6)	0.152 (7)	0.094 (6)	0.060 (5)	0.000 (5)	−0.008 (5)
C46	0.141 (7)	0.147 (6)	0.107 (6)	0.082 (6)	0.020 (6)	0.015 (5)
C47	0.137 (8)	0.208 (10)	0.106 (7)	0.084 (7)	0.018 (6)	0.000 (6)
C48	0.197 (9)	0.218 (8)	0.118 (6)	0.125 (8)	−0.005 (6)	0.009 (6)
C49	0.212 (8)	0.273 (8)	0.146 (6)	0.134 (8)	−0.011 (6)	0.003 (6)
C50	0.248 (10)	0.291 (9)	0.162 (8)	0.137 (8)	−0.038 (6)	0.002 (7)
C51	0.294 (12)	0.301 (11)	0.205 (10)	0.105 (9)	−0.034 (8)	0.016 (8)
C52	0.302 (16)	0.275 (12)	0.300 (16)	0.141 (10)	0.016 (12)	−0.015 (11)

### Geometric parameters (Å, º)

**Table:**

O1—C2	1.387 (6)	C25—C28	1.516 (7)
O1—H1	0.822 (11)	C26—C27	1.391 (7)
O2—C5	1.396 (5)	C27—H27	0.9500
O2—C36	1.426 (7)	C28—C29	1.538 (7)
O3—C9	1.387 (6)	C28—H28A	0.9900
O3—C37	1.426 (7)	C28—H28B	0.9900
O4—C12	1.395 (6)	C29—C30	1.401 (7)
O4—C38	1.423 (7)	C29—C34	1.411 (7)
O5—C16	1.408 (6)	C30—C31	1.411 (7)
O5—C39	1.421 (7)	C31—C32	1.407 (7)
O6—C19	1.387 (6)	C31—H31	0.9500
O6—C40	1.422 (6)	C32—C33	1.394 (7)
O7—C23	1.390 (6)	C32—C35	1.531 (7)
O7—C41	1.411 (7)	C33—C34	1.392 (7)
O8—C26	1.414 (6)	C34—H34	0.9500
O8—C42	1.423 (7)	C35—H35A	0.9900
O9—C30	1.396 (6)	C35—H35B	0.9900
O9—C43	1.447 (7)	C36—H36A	0.9800
O10—C33	1.396 (6)	C36—H36B	0.9800
O10—C44	1.434 (7)	C36—H36C	0.9800
O11—H11A	0.827 (10)	C37—H37A	0.9800
O11—H11B	0.823 (10)	C37—H37B	0.9800
N1—C45	1.403 (9)	C37—H37C	0.9800
N1—H1B	0.9100 (13)	C38—H38A	0.9800
N1—H1A	0.9102 (12)	C38—H38B	0.9800
C1—C6	1.403 (6)	C38—H38C	0.9800
C1—C2	1.413 (7)	C39—H39A	0.9800
C1—C35	1.528 (6)	C39—H39B	0.9800
C2—C3	1.404 (6)	C39—H39C	0.9800
C3—C4	1.374 (6)	C40—H40A	0.9800
C3—H3	0.9500	C40—H40B	0.9800
C4—C5	1.412 (7)	C40—H40C	0.9800
C4—C7	1.530 (6)	C41—H41A	0.9800
C5—C6	1.425 (7)	C41—H41B	0.9800
C6—H6	0.9500	C41—H41C	0.9800
C7—C8	1.528 (7)	C42—H42A	0.9800
C7—H7A	0.9900	C42—H42B	0.9800
C7—H7B	0.9900	C42—H42C	0.9800
C8—C9	1.401 (7)	C43—H43A	0.9800
C8—C13	1.419 (7)	C43—H43B	0.9800
C9—C10	1.414 (7)	C43—H43C	0.9800
C10—C11	1.405 (7)	C44—H44A	0.9800
C10—H10	0.9500	C44—H44B	0.9800
C11—C12	1.402 (7)	C44—H44C	0.9800
C11—C14	1.534 (7)	C45—C46	1.448 (10)
C12—C13	1.383 (7)	C45—H45A	0.9900
C13—H13	0.9500	C45—H45B	0.9900
C14—C15	1.532 (7)	C46—C47	1.428 (10)
C14—H14A	0.9900	C46—H46A	0.9900
C14—H14B	0.9900	C46—H46B	0.9900
C15—C20	1.400 (7)	C47—C48	1.486 (12)
C15—C16	1.420 (7)	C47—H47A	0.9900
C16—C17	1.378 (7)	C47—H47B	0.9900
C17—C18	1.412 (7)	C48—C49	1.429 (13)
C17—H17	0.9500	C48—H48A	0.9900
C18—C19	1.397 (7)	C48—H48B	0.9900
C18—C21	1.519 (7)	C49—C50	1.529 (8)
C19—C20	1.409 (7)	C49—H49A	0.9900
C20—H20	0.9500	C49—H49B	0.9900
C21—C22	1.526 (7)	C50—C51	1.505 (9)
C21—H21A	0.9900	C50—H50A	0.9900
C21—H21B	0.9900	C50—H50B	0.9900
C22—C27	1.394 (7)	C51—C52	1.512 (9)
C22—C23	1.408 (7)	C51—H51A	0.9900
C23—C24	1.409 (7)	C51—H51B	0.9900
C24—C25	1.387 (7)	C52—H52A	0.9800
C24—H24	0.9500	C52—H52B	0.9800
C25—C26	1.405 (7)	C52—H52C	0.9800
			
C2—O1—H1	109 (8)	C30—C31—H31	119.2
C5—O2—C36	118.7 (4)	C33—C32—C31	117.6 (5)
C9—O3—C37	117.5 (5)	C33—C32—C35	122.4 (5)
C12—O4—C38	117.8 (5)	C31—C32—C35	119.9 (5)
C16—O5—C39	118.3 (5)	C34—C33—C32	120.7 (5)
C19—O6—C40	119.1 (4)	C34—C33—O10	123.6 (5)
C23—O7—C41	117.6 (4)	C32—C33—O10	115.7 (5)
C26—O8—C42	118.3 (4)	C33—C34—C29	122.8 (5)
C30—O9—C43	117.9 (4)	C33—C34—H34	118.6
C33—O10—C44	119.2 (5)	C29—C34—H34	118.6
H11A—O11—H11B	103.8 (17)	C1—C35—C32	112.3 (4)
C45—N1—H1B	118.2 (13)	C1—C35—H35A	109.1
C45—N1—H1A	118.1 (13)	C32—C35—H35A	109.1
H1B—N1—H1A	95.8 (10)	C1—C35—H35B	109.1
C6—C1—C2	118.0 (4)	C32—C35—H35B	109.1
C6—C1—C35	120.7 (5)	H35A—C35—H35B	107.9
C2—C1—C35	121.4 (4)	O2—C36—H36A	109.5
O1—C2—C3	122.5 (5)	O2—C36—H36B	109.5
O1—C2—C1	117.9 (4)	H36A—C36—H36B	109.5
C3—C2—C1	119.6 (4)	O2—C36—H36C	109.5
C4—C3—C2	123.9 (5)	H36A—C36—H36C	109.5
C4—C3—H3	118.0	H36B—C36—H36C	109.5
C2—C3—H3	118.0	O3—C37—H37A	109.5
C3—C4—C5	116.7 (4)	O3—C37—H37B	109.5
C3—C4—C7	122.6 (5)	H37A—C37—H37B	109.5
C5—C4—C7	120.6 (4)	O3—C37—H37C	109.5
O2—C5—C4	116.5 (4)	H37A—C37—H37C	109.5
O2—C5—C6	122.4 (5)	H37B—C37—H37C	109.5
C4—C5—C6	121.1 (4)	O4—C38—H38A	109.5
C1—C6—C5	120.7 (5)	O4—C38—H38B	109.5
C1—C6—H6	119.6	H38A—C38—H38B	109.5
C5—C6—H6	119.6	O4—C38—H38C	109.5
C8—C7—C4	111.9 (4)	H38A—C38—H38C	109.5
C8—C7—H7A	109.2	H38B—C38—H38C	109.5
C4—C7—H7A	109.2	O5—C39—H39A	109.5
C8—C7—H7B	109.2	O5—C39—H39B	109.5
C4—C7—H7B	109.2	H39A—C39—H39B	109.5
H7A—C7—H7B	107.9	O5—C39—H39C	109.5
C9—C8—C13	117.1 (5)	H39A—C39—H39C	109.5
C9—C8—C7	121.3 (5)	H39B—C39—H39C	109.5
C13—C8—C7	121.5 (5)	O6—C40—H40A	109.5
O3—C9—C8	116.8 (5)	O6—C40—H40B	109.5
O3—C9—C10	122.5 (5)	H40A—C40—H40B	109.5
C8—C9—C10	120.7 (5)	O6—C40—H40C	109.5
C11—C10—C9	121.2 (5)	H40A—C40—H40C	109.5
C11—C10—H10	119.4	H40B—C40—H40C	109.5
C9—C10—H10	119.4	O7—C41—H41A	109.5
C12—C11—C10	118.1 (5)	O7—C41—H41B	109.5
C12—C11—C14	121.9 (5)	H41A—C41—H41B	109.5
C10—C11—C14	120.0 (5)	O7—C41—H41C	109.5
C13—C12—O4	123.2 (5)	H41A—C41—H41C	109.5
C13—C12—C11	120.7 (5)	H41B—C41—H41C	109.5
O4—C12—C11	116.1 (5)	O8—C42—H42A	109.5
C12—C13—C8	122.3 (5)	O8—C42—H42B	109.5
C12—C13—H13	118.8	H42A—C42—H42B	109.5
C8—C13—H13	118.8	O8—C42—H42C	109.5
C15—C14—C11	112.3 (4)	H42A—C42—H42C	109.5
C15—C14—H14A	109.1	H42B—C42—H42C	109.5
C11—C14—H14A	109.1	O9—C43—H43A	109.5
C15—C14—H14B	109.1	O9—C43—H43B	109.5
C11—C14—H14B	109.1	H43A—C43—H43B	109.5
H14A—C14—H14B	107.9	O9—C43—H43C	109.5
C20—C15—C16	116.5 (5)	H43A—C43—H43C	109.5
C20—C15—C14	121.5 (5)	H43B—C43—H43C	109.5
C16—C15—C14	122.0 (5)	O10—C44—H44A	109.5
C17—C16—O5	125.5 (5)	O10—C44—H44B	109.5
C17—C16—C15	120.4 (5)	H44A—C44—H44B	109.5
O5—C16—C15	114.2 (5)	O10—C44—H44C	109.5
C16—C17—C18	123.2 (5)	H44A—C44—H44C	109.5
C16—C17—H17	118.4	H44B—C44—H44C	109.5
C18—C17—H17	118.4	N1—C45—C46	119.9 (7)
C19—C18—C17	116.9 (5)	N1—C45—H45A	107.3
C19—C18—C21	122.2 (5)	C46—C45—H45A	107.3
C17—C18—C21	120.8 (4)	N1—C45—H45B	107.3
O6—C19—C18	116.3 (5)	C46—C45—H45B	107.3
O6—C19—C20	123.6 (4)	H45A—C45—H45B	106.9
C18—C19—C20	120.1 (5)	C47—C46—C45	122.9 (8)
C15—C20—C19	122.9 (5)	C47—C46—H46A	106.6
C15—C20—H20	118.6	C45—C46—H46A	106.6
C19—C20—H20	118.6	C47—C46—H46B	106.6
C18—C21—C22	113.7 (4)	C45—C46—H46B	106.6
C18—C21—H21A	108.8	H46A—C46—H46B	106.6
C22—C21—H21A	108.8	C46—C47—C48	119.4 (8)
C18—C21—H21B	108.8	C46—C47—H47A	107.5
C22—C21—H21B	108.8	C48—C47—H47A	107.5
H21A—C21—H21B	107.7	C46—C47—H47B	107.5
C27—C22—C23	117.5 (5)	C48—C47—H47B	107.5
C27—C22—C21	121.0 (4)	H47A—C47—H47B	107.0
C23—C22—C21	121.4 (5)	C49—C48—C47	120.8 (9)
O7—C23—C22	116.6 (5)	C49—C48—H48A	107.1
O7—C23—C24	123.6 (5)	C47—C48—H48A	107.1
C22—C23—C24	119.7 (5)	C49—C48—H48B	107.1
C25—C24—C23	122.8 (5)	C47—C48—H48B	107.1
C25—C24—H24	118.6	H48A—C48—H48B	106.8
C23—C24—H24	118.6	C48—C49—C50	119.8 (11)
C24—C25—C26	116.8 (5)	C48—C49—H49A	107.4
C24—C25—C28	120.6 (4)	C50—C49—H49A	107.4
C26—C25—C28	122.5 (5)	C48—C49—H49B	107.4
C27—C26—C25	121.2 (5)	C50—C49—H49B	107.4
C27—C26—O8	124.3 (5)	H49A—C49—H49B	106.9
C25—C26—O8	114.4 (5)	C51—C50—C49	123.4 (13)
C26—C27—C22	122.0 (5)	C51—C50—H50A	106.5
C26—C27—H27	119.0	C49—C50—H50A	106.5
C22—C27—H27	119.0	C51—C50—H50B	106.5
C25—C28—C29	110.9 (4)	C49—C50—H50B	106.5
C25—C28—H28A	109.5	H50A—C50—H50B	106.5
C29—C28—H28A	109.5	C50—C51—C52	130.4 (16)
C25—C28—H28B	109.5	C50—C51—H51A	104.7
C29—C28—H28B	109.5	C52—C51—H51A	104.7
H28A—C28—H28B	108.0	C50—C51—H51B	104.7
C30—C29—C34	116.6 (5)	C52—C51—H51B	104.7
C30—C29—C28	122.4 (5)	H51A—C51—H51B	105.7
C34—C29—C28	121.1 (5)	C51—C52—H52A	109.5
O9—C30—C29	115.1 (5)	C51—C52—H52B	109.5
O9—C30—C31	124.0 (5)	H52A—C52—H52B	109.5
C29—C30—C31	120.9 (5)	C51—C52—H52C	109.5
C32—C31—C30	121.5 (5)	H52A—C52—H52C	109.5
C32—C31—H31	119.2	H52B—C52—H52C	109.5
			
C6—C1—C2—O1	−179.9 (4)	C16—C15—C20—C19	3.5 (7)
C35—C1—C2—O1	−1.1 (7)	C14—C15—C20—C19	−176.3 (4)
C6—C1—C2—C3	−0.3 (7)	O6—C19—C20—C15	177.5 (5)
C35—C1—C2—C3	178.5 (5)	C18—C19—C20—C15	−2.9 (8)
O1—C2—C3—C4	178.3 (5)	C19—C18—C21—C22	−90.5 (6)
C1—C2—C3—C4	−1.3 (8)	C17—C18—C21—C22	85.6 (6)
C2—C3—C4—C5	1.6 (7)	C18—C21—C22—C27	91.1 (6)
C2—C3—C4—C7	−176.4 (5)	C18—C21—C22—C23	−87.4 (6)
C36—O2—C5—C4	−178.5 (5)	C41—O7—C23—C22	−177.5 (6)
C36—O2—C5—C6	0.6 (7)	C41—O7—C23—C24	3.5 (9)
C3—C4—C5—O2	178.7 (4)	C27—C22—C23—O7	−179.4 (4)
C7—C4—C5—O2	−3.3 (7)	C21—C22—C23—O7	−0.8 (7)
C3—C4—C5—C6	−0.4 (7)	C27—C22—C23—C24	−0.3 (7)
C7—C4—C5—C6	177.6 (4)	C21—C22—C23—C24	178.3 (4)
C2—C1—C6—C5	1.4 (7)	O7—C23—C24—C25	178.1 (5)
C35—C1—C6—C5	−177.4 (4)	C22—C23—C24—C25	−0.9 (8)
O2—C5—C6—C1	179.8 (4)	C23—C24—C25—C26	1.1 (7)
C4—C5—C6—C1	−1.1 (7)	C23—C24—C25—C28	−176.3 (4)
C3—C4—C7—C8	93.5 (6)	C24—C25—C26—C27	−0.1 (7)
C5—C4—C7—C8	−84.4 (6)	C28—C25—C26—C27	177.3 (4)
C4—C7—C8—C9	−85.0 (6)	C24—C25—C26—O8	−179.7 (4)
C4—C7—C8—C13	91.6 (5)	C28—C25—C26—O8	−2.3 (7)
C37—O3—C9—C8	153.9 (5)	C42—O8—C26—C27	−14.8 (8)
C37—O3—C9—C10	−26.7 (7)	C42—O8—C26—C25	164.8 (5)
C13—C8—C9—O3	−179.6 (4)	C25—C26—C27—C22	−1.2 (8)
C7—C8—C9—O3	−2.8 (6)	O8—C26—C27—C22	178.3 (5)
C13—C8—C9—C10	1.0 (7)	C23—C22—C27—C26	1.4 (7)
C7—C8—C9—C10	177.8 (4)	C21—C22—C27—C26	−177.2 (4)
O3—C9—C10—C11	179.0 (4)	C24—C25—C28—C29	82.2 (6)
C8—C9—C10—C11	−1.7 (7)	C26—C25—C28—C29	−95.1 (5)
C9—C10—C11—C12	1.4 (7)	C25—C28—C29—C30	−90.2 (5)
C9—C10—C11—C14	−176.9 (4)	C25—C28—C29—C34	87.3 (5)
C38—O4—C12—C13	9.0 (8)	C43—O9—C30—C29	−179.0 (4)
C38—O4—C12—C11	−173.3 (5)	C43—O9—C30—C31	0.2 (7)
C10—C11—C12—C13	−0.6 (7)	C34—C29—C30—O9	179.8 (4)
C14—C11—C12—C13	177.7 (4)	C28—C29—C30—O9	−2.5 (6)
C10—C11—C12—O4	−178.3 (4)	C34—C29—C30—C31	0.7 (6)
C14—C11—C12—O4	0.0 (7)	C28—C29—C30—C31	178.3 (4)
O4—C12—C13—C8	177.5 (4)	O9—C30—C31—C32	−179.8 (4)
C11—C12—C13—C8	0.0 (7)	C29—C30—C31—C32	−0.7 (7)
C9—C8—C13—C12	−0.2 (7)	C30—C31—C32—C33	0.0 (6)
C7—C8—C13—C12	−177.0 (4)	C30—C31—C32—C35	−178.3 (4)
C12—C11—C14—C15	−94.5 (5)	C31—C32—C33—C34	0.7 (7)
C10—C11—C14—C15	83.8 (6)	C35—C32—C33—C34	179.0 (4)
C11—C14—C15—C20	96.3 (6)	C31—C32—C33—O10	179.9 (4)
C11—C14—C15—C16	−83.4 (6)	C35—C32—C33—O10	−1.8 (6)
C39—O5—C16—C17	−9.9 (8)	C44—O10—C33—C34	2.3 (7)
C39—O5—C16—C15	169.1 (5)	C44—O10—C33—C32	−176.9 (5)
C20—C15—C16—C17	−2.9 (7)	C32—C33—C34—C29	−0.8 (7)
C14—C15—C16—C17	176.8 (5)	O10—C33—C34—C29	−179.9 (4)
C20—C15—C16—O5	178.0 (4)	C30—C29—C34—C33	0.0 (7)
C14—C15—C16—O5	−2.3 (7)	C28—C29—C34—C33	−177.6 (4)
O5—C16—C17—C18	−179.1 (5)	C6—C1—C35—C32	84.2 (6)
C15—C16—C17—C18	1.9 (8)	C2—C1—C35—C32	−94.6 (6)
C16—C17—C18—C19	−1.2 (8)	C33—C32—C35—C1	−84.7 (6)
C16—C17—C18—C21	−177.5 (5)	C31—C32—C35—C1	93.5 (5)
C40—O6—C19—C18	−168.9 (5)	N1—C45—C46—C47	175.8 (8)
C40—O6—C19—C20	10.7 (8)	C45—C46—C47—C48	−179.2 (9)
C17—C18—C19—O6	−178.8 (4)	C46—C47—C48—C49	172.4 (10)
C21—C18—C19—O6	−2.5 (7)	C47—C48—C49—C50	179.8 (10)
C17—C18—C19—C20	1.6 (7)	C48—C49—C50—C51	103.8 (18)
C21—C18—C19—C20	177.9 (4)	C49—C50—C51—C52	30 (3)

### Hydrogen-bond geometry (Å, º)

**Table:**

*D*—H···*A*	*D*—H	H···*A*	*D*···*A*	*D*—H···*A*
O11—H11*A*···N1	0.83 (2)	1.98 (5)	2.770 (10)	159 (7)
O11—H11*B*···O1	0.80 (10)	2.40 (10)	3.060 (10)	145 (10)
O1—H1···O11^i^	0.82 (5)	1.90 (5)	2.711 (7)	168 (9)

Symmetry code: (i) −*x*+2, −*y*+1, −*z*+1.
